# First Rwanda National Trauma Symposium 2019: Challenges and priorities

**DOI:** 10.7189/jogh.10.010201

**Published:** 2020-06

**Authors:** Ashley Rosenberg, Faustin Ntirenganya, Irene Bagahirwa, Gabin Mbanjumucyo, Lambert Rutayisire, Severien Muneza, Innocent Nzeyimana, Eric Benimana, Ernest Nahayo, Busisiwe Bhengu, Assuman Nuhu, Arsene Muhumuza, Costica Uwitonze, Ghislaine Umwali, Menelas Nkeshimana, Jeanne D’Arc Nyinawankusi, Elizabeth Krebs, Jean Marie Uwitonze, Igance Kabagema, Theophile Dushime, Jean Claude Byiringiro, Gilles Ndayisaba, Sudha Jayaraman, on behalf of the participants for the first national Rwanda Trauma Symposium 2019 in Kigali, Rwanda

**Affiliations:** 1 Virginia Commonwealth University Department of Surgery, Richmond, Virginia, USA; 2 University Teaching Hospital of Kigali Department of Surgery, Kigali, Rwanda; 3 Rwanda Biomedical Center, Kigali, Rwanda; 4 University Teaching Hospital of Kigali Department of Accident and Emergency, Kigali, Rwanda; 5 Rwanda National Police, Kigali, Rwanda; 6 Rwanda Military Hospital Department of Accident and Emergency, Kigali, Rwanda; 7 University of Rwanda School of Nursing, Kigali, Rwanda; 8 University of Rwanda College of Medicine and Health Sciences, Kigali, Rwanda; 9 Rwanda Association for Biomedical Engineering, Kigali, Rwanda; 10 Collaboration for Evidence-based Healthcare and Public Health in Africa, Kigali, Rwanda; 11 Service d’Aide Medicale Urgente- Rwanda Ministry of Health, Kigali, Rwanda; 12 Thomas Jefferson University, Sidney Kimmel Medical College, Philadelphia, Pennsylvania, USA; 13 Rwanda Biomedical Center, Department of Noncommunicable Diseases, Kigali, Rwanda; 14 Virginia Commonwealth University Program for Global Surgery, Richmond, Virginia, USA; * Joint first authorship.

The global burden of trauma is enormous. Worldwide, nearly 5 million people die from trauma every year [1]. Trauma accounts for 9% of global mortality, which is 1.7 times the number of deaths from HIV/AIDS, tuberculosis and malaria, combined [2]. Trauma deaths are on the rise. The three-leading trauma-related causes of death are predicted to be among the leading 20 causes of death worldwide by 2030 [3]. In both low-middle income (LMIC) and high-income (HIC) countries, road traffic injuries (RTI) rank as a leading cause of death for 5-44 year-olds [1]. In addition, tens of millions of people suffer from injury-related disability leading to increased utilization of health care, an already limited resource in LMICs [4]. The most common demographic to suffer from an RTI are males in the most productive years of their lives [5]. The impact of these injured males impacts the entire family unit and community. The reasons that LMICs face so much disability and more than 90% of global trauma-related deaths are complex, including limited prehospital and emergency care, inadequate rehabilitation systems, on top of delicate social welfare and economic foundations [6-8].

Despite this evidence, efforts aimed at addressing the burden of trauma have been minimal. Injuries have been neglected from the global health agenda despite being predictable and largely preventable [4]. At the country level, despite the significant burden of disease, per capita yearly expenditure on road safety has been negligible (0.07-0.09USD) even though trauma prevention safety measures have been shown to be cost-effective [9-11].

Recognizing the burden and the inadequate policy response, the UN General Assembly adopted Resolution 72/271 on Improving Global Road Safety in April 2018 [12,13]. In addition, the UN Sustainable Development Goals (SDG) have incorporated a major emphasis on trauma care and prevention including: targeting a reduction in global road traffic deaths and injuries by 50% by 2020 (3.6); ending violence against women and girls (5.2), providing safe, sustainable transport (11.2); and reducing violence everywhere (16.1), and ending violence against children (16.2) [14].

The momentum from these global policy stances, has led to concerted attempts to study trauma in the African continent. The East African Injury Symposium, an effort by the Johns Hopkins School of Public Health and Makerere University was held in Uganda March 2018, and the first African Road Safety forum held in Morocco in 2018 to promote the trauma-related SDGs [15,16].

The Ministry of Health of Rwanda, which previously led major achievements in the MDGs, has recognized and supported this focus on trauma through multiple transnational collaborations. They co-hosted the first Rwanda National Trauma Symposium in February 2019 in partnership with Virginia Commonwealth University and support from the US National Institutes of Health’s National Cancer Institute and the Fogarty International Center to bring together a diverse set of stakeholders who are involved in trauma care, management, prevention, policy and research locally. This paper summarizes the objectives, discussion and recommendations derived from the first Rwanda National Trauma Symposium and aims to provide sufficient detail so that other LMICs may use this as a tool to design their own initiatives.

## ORGANIZING TEAM

Members included representatives from the Rwanda Ministry of Health, trauma related non-governmental organizations (NGOs), representatives from the largest university teaching hospital (emergency medicine, general surgery, orthopedic surgery, neurosurgery), Emergency Medical Services, the University of Rwanda, and foreign institution representatives from VCU.

A consensus on need, structure and topics was developed by this team after discussion across a diverse group of colleagues and institutions. Logistics and coordination including promotional materials, website and registration links were determined. The event was funded through the NIH P20 grant managed by a local contract research organization, Eagle Research Center and the Rwanda Biomedical Center.

The organizing team discussed the need for the symposium and its potential structure. Permission was obtained to use official logos of the Ministry of Health of Rwanda, Virginia Commonwealth University, and the National Institutes of Health for flyers, banners, and agendas. A large push for marketing on social media via Twitter and Instagram was made to solicit interested participants and speakers. Staff from hospitals and health-related NGOs were contacted. Online registration was created which also included several questions relating to trauma and research in Rwanda.

## PARTICIPANTS

The conference drew more than 100 participants and researchers from various sectors, including the Rwanda Ministry of Health, Rwanda National Police, local and international NGOs, USAID, the University of Rwanda College of Medicine and Health Sciences, and medical doctors and nurses from various provincial and district hospitals in Rwanda and on its borders. Additionally, several researchers and educators from the United States and Europe as well as the American Ambassador to Rwanda attended the conference. Conference registrants included 48 medical students (26%). 37 nurses (20%), 28 medical doctors (15%), 9 research assistants (5%), 7 residents (4%), 6 teachers/lecturers (3%), and 6 NGO workers (6%).

In order to design the symposium to be responsive to ongoing local work, upon registering, participants were asked for their opinions on four topics in free text format:

1. The single greatest challenge in trauma management in Rwanda,
2. The main research priority for trauma in Rwanda,
3. The main area of trauma-related work they were involved in,
4. What, if any, collaboration they were part of?

## SYMPOSIUM OBJECTIVES

The conference objectives as identified by the organizing team were 3-fold:

- Identifying current challenges in trauma care in Rwanda,
- Identifying current trauma collaborations in Rwanda,
- Identifying trauma-related research priorities in Rwanda.

## PARTICIPANT RESPONSES

When asked about the single greatest challenge in trauma management in Rwanda, participants described challenges of logistical and human resource constraints as well as limited research experience (**Table 1**). When asked about the main research priority for trauma, participants responded about education, patient outcomes and access to care (**Table 2**). Responding participants included surgeons (orthopedic, neuro, general, dental), emergency physicians, radiologists, critical care providers, nurses, physiotherapists, medical students, prehospital staff, research assistants, Red Cross workers, and academic lecturers. When asked with whom participants collaborate with in their trauma related work, 39 physicians at 10 hospitals and three trauma-related NGOs were mentioned.

**Table 1:** Survey responses for the themes for greatest challenge in trauma management

**Resources:**
Lack of resources
Lack of intensive care unit (ICU) beds
Lack of health care providers/surgeons
Infrastructure
**Research:**
Trauma registry
Lack of research
Education:
Lack of standardized protocols
Lack of formal prehospital courses
Lack of Advanced Trauma Life Support (ATLS) training
Trauma prevention
**In hospital:**
Inability to care for trauma patients at the district hospital level
Delays in care
Burns
Triage
Referral system
Motor vehicle crashes

**Table 2:** Survey responses for the teams for main research priority for trauma

**Outcomes:**
Late complications of trauma
Mortality rate
Outcomes of trauma patients
Post-surgical infection
Trauma-related infection
**Access to care:**
Access to trauma care:
Prehospital care
Ambulance response
**Education:**
Injury prevention
Effect of increased trauma training
**Causes:**
Head and spinal trauma injuries
Causes of trauma
**In hospital care:**
Management of trauma
Referral hospitals

## THE CONFERENCE STRUCTURE AND PROCEEDINGS

The conference was a one-day event with four sessions. The first session was a broad overview of current challenges in trauma care in Rwanda. Seven presentations were given and focused on challenges related to trauma/injury care at the Rwanda prehospital emergency service (SAMU), the Rwanda National Police, emergency medicine, orthopedic surgery and neurosurgery from University Teaching Hospital of Kigali (CHUK), the Rwanda Biomedical Center. The second session detailed some specific responses to current challenges in trauma care in Rwanda. Four presentations were given and focused on trauma at the Rwanda Military Hospital, sports related trauma, prehospital injuries and the role of the advanced practice nurse.

Next, three parallel breakout sessions were moderated by members of the organizing team that addressed three broad categories of topics assembled from the symposium registration questions: challenges to systems, challenges to hospitals, and challenges in patient outcomes. The group discussing challenges to systems reviewed prehospital care, delays in care, access to care, referral system, community education and trauma care in district hospitals. The challenges to hospitals group cited the need for more health care providers, the need for manuals, standardized protocols, resources, education and Advanced Trauma Life Support (ATLS) training. The challenges for outcomes group reported limited data, trauma registries, surgical infections and the effectiveness of current training programs.

The fourth session discussed current trauma collaborations in Rwanda and featured a discussion of research priorities and plans for the future. Six unique collaborations were discussed, ranging from NIH/Fogarty-funded collaborations to Collaboration for Evidence-based Healthcare and Public Health in Africa (CEBHA plus) - a collaboration between the German Federal Ministry of Education and Research, independent global health initiatives and universities in five Sub-Saharan African countries. In addition, two unique projects for trauma and prehospital care dispatch were discussed.

The event was opened by the Director General of Clinical Services from the Ministry of Health of Rwanda and a collaborative discussion on the next steps was moderated by the Director of Injury Unit at RBC along with colleagues from Virginia Commonwealth University. The Division Manager of SAMU/MOH concluded the event. Lunch and breaks gave participants an opportunity to discuss ideas and form connections in order to work together in the future.

## SUMMARY OF SYMPOSIUM BY TOPIC AREAS

### Prehospital trauma and EMS systems

The current state of prehospital care in Rwanda was discussed by the leadership of the Service d’Aide Medicale Urgente (SAMU). SAMU coordinates 12 ambulances in Kigali and 273 at the provincial/district hospital level and also has the capacity to operate air ambulances and a boat ambulance on Lake Kivu. SAMU manages national dispatch using “912”. The speaker discussed some of the challenges SAMU faces, including limited EMS training, limited resources, and inefficient coordination and communication across the EMS system. Strengths highlighted included the existence of the toll-free 912 number for emergencies and a national network for emergency services and critical care and trauma nursing programs. SAMU usage data was presented and showed that a majority of cases were due to road traffic crashes (RTCs) [17].

A formal collaboration between the Ministry of Health of Rwanda (MOH) and Virginia Commonwealth University (VCU), through a Memorandum of Understanding to facilitate trauma and emergency medical systems development was also discussed. Highlights included funding for the program through several sources which allowed SAMU and other professionals to spend time in Virginia learning about emergency medical systems. Additionally, a Rotary Foundation led train-the-trainer education program for standardizing prehospital trauma, medical, pediatric, obstetric and neonatal care was discussed. SAMU staff developed an instructor core and taught staff from district and provincial hospitals through this program. Through another grant, the SAMU and VCU teams also created a series of protocols and checklists for the most common conditions seen and treated by SAMU teams and the MOH have approved these the national standard for SAMU. Implementation is in progress.

Two innovative mobile application and online service concepts for the prehospital and transfer between hospitals were also discussed. One proposed a 912 online ambulance service in collaboration with RwandaBuild, a local software accelerator and VCU, which would provide GPS and directions to the ambulance service and a communication platform for dispatch, ambulance and emergency medical doctors throughout Kigali. Another proposed a mobile application that connected users at health centers, district hospitals and tertiary hospitals and would make it easy to share patient information and data across hospitals for support and/or transfer. Both speakers were in the process of seeking funding support to move their concepts forward.

### Clinical trauma care

Various clinicians presented on the state of trauma care in Rwanda and current challenges in the clinical setting. The Head of Emergency at both the University Teaching Hospital of Kigali (CHUK) and the Rwanda Military Hospital (RMH) presented the status at their facilities. At RMH, trauma was responsible for nearly one third of all patient admissions from the Emergency Department – the majority being male in the 21-40 age range followed by school-aged children. Common trauma patterns were noted to be mostly lower limb injuries followed by upper limb injuries. Orthopedic trauma care at CHUK including the large amount of fractures resulting from road traffic crashes was discussed. Challenges faced include the large number of cases, few hospitals able to handle trauma, and the limited number of surgeons (15) performing orthopedic surgery in the country all leading to delays in presentation after trauma and long wait times [18]. A residency program has been created at CHUK to increase the human resources, but more infrastructure is needed. Representatives from the CHUK and RMH neurosurgery departments discussed the high prevalence of traumatic brain injury (TBI) in Rwanda, representing 50% of all trauma cases at CHUK. Patient epidemiology focused on road traffic incidents and assaults as the main mechanism of injury for TBI patients. Bicycle riders were cited as having a high incidence of TBI. Challenges include a severe shortage of in-country neurosurgeons (n = 6), rising number of TBIs seeking neurosurgery care, and a shortage of intensive care unit (ICU) beds leading to long waiting times for patients.

The new system of triage at CHUK which has redesigned the flow of the emergency department was discussed along with patient flow times [19]. The new emergency medicine (EM) residency through the Human Resources for Health Program [20], allowing for the expansion of EM care in Rwanda, and the first graduating class of five EM residents were mentioned. Some challenges raised included overcrowding, patient length of stay in the emergency department, and delay in disposition. An innovative WhatsApp-based communication group called ED SITREP has been created at CHUK to discuss bed management in real time and on regular intervals to support efficient use of available resources.

Other speakers included a physiotherapist who discussed the high burden of sports-related injuries in Rwanda, the management of these conditions and challenges such as lack of medical support, rehabilitation and financial resources.

A nurse educator discussed the potential for addressing the shortage of physician workforce through task shifting and as a mechanism to treat more patients and those with higher complexity and acuity [21]. She outlined a strategic plan for nurse practitioner role implementation with a set of regulatory requirements and structured training.

### Trauma prevention, research and policy

Various speakers discussed ongoing trauma prevention efforts in Rwanda. The Director of the Injury Unit at RBC presented on the Unit’s coordination of trauma-related interventions in the country. A MOH-VCU-WHO-NIH collaborative trauma registry was presented. The registry was proposed to address Sustainable Development Goal 3.6 reducing the numbers of global deaths and injuries from Motor Vehicle Crashes by half by 2030. The registry is part of an MOU between VCU and the MOH of Rwanda to facilitate capacity for injury and emergency care in the country. The registry will build on an existing pilot registry at a number of hospitals in Rwanda. Data will be captured using a standardized data collection tool from WHO’s international trauma registry platform. After 2 years, the registry will be transferred to the Ministry of Health and managed through the division of injuries and function as a permanent injury surveillance registry that informs the MOH of Rwanda of the burden of injury in Rwanda and allows local priority setting. A lead representative from the Collaboration for Evidence-Based Healthcare and Public Health in Africa (CEBHA+) presented about their role in providing high quality data to assess the effectiveness of interventions and upcoming projects [22]. A Thomas Jefferson University injury researcher presented on another interdisciplinary collaboration between multiple private, public and academic Rwandan partners that detailed their in-depth analyses of motorcycle crashes, the helmets worn in the crash and the injuries sustained. Their team introduced the concept of potential design innovations and a made-in-Rwanda motorcycle helmet. Infrastructure and health facility design for provision of emergency services was discussed by an engineer who presented on considerations for improving the design of emergency departments as well as the need for resuscitation, surgery and triage areas in emergency departments.

The day concluded with talks on trauma prevention and policy. A spokesperson for the Rwanda National Police presented on road safety and the role of the police in trauma care and opportunities to strengthen the working relationship between the prehospital ambulance service and the police.

The leaders of two NGOs – Healthy People Rwanda (HPR) and the Rwanda Red Cross also presented summaries of their efforts. HPR aims to reduce morbidity and mortality from road traffic crashes, especially among children and youth in Rwanda via training sessions for students and young drivers in road safety and first aid, and integration of these topics into school curriculum. The Rwanda Red Cross team has created a first aid manual for children and first responders and is working on an EMT training manual. They discussed their presence during natural disasters and at mass gatherings, meetings and sports events and the expertise they have available to assist in such settings.

### Recommendations and next steps for trauma care in Rwanda

Three working groups developed priorities for trauma care in Rwanda. Participants then gathered and developed a consensus of 33 recommendations and plans as a group for trauma care in Rwanda going forward. Recommendations included broad themes of medical education/protocols, public education, research, prehospital and in-hospital care (**Table 3**).

**Table 3:** Recommendations for trauma care in Rwanda

**Medical education:**
Develop national guidelines on trauma care
Create protocols and standards for district hospitals
Capacity build through courses for non-physicians
Educate and equip community health workers
Provide team courses for health care providers to work together
**Community/public education:**
Tackle primary trauma prevention education in the community
Increase public awareness on basic emergency lifesaving skills
Train motorcycle drivers in first aid and basic life support
Place first aid kits on motorcycles, bicycles, vehicles and buses
Educate the public on the proper use of helmets
**Research:**
Establish a national trauma registry
**Prehospital:**
Improve GPS coordinates to facilitate users getting to the scene
Improve prehospital care of injured patients during transporters
Ease communication among respondents at the scene
Create more centralized dispatch
Improve policies to help ambulances struggling in traffic jams
Strengthen the connection with MedEvac
**In hospital:**
Increase trauma care management at all levels of the health care system
Upgrade technologies and infrastructure requirements for trauma care
Optimize of referral procedures that delay patient management
Require private hospitals to have emergency departments
Task shift non-physicians to cover the gap
Restructure emergency department (ED) patient admissions to avoid delays in care
Improve the design of health facilities to make it more applicable for resuscitations, surgery and triage and to have enough space for patients

The conference participants described current deficiencies in medical education. They cited a lack of formal protocols to guide trauma care starting with the prehospital level all the way to patient rehabilitation. Due to the critical lack of physicians, participants stressed the importance of increased education and support for non-physicians such as community health workers at the community level to nurses in hospital stressing the educational benefits of task shifting. Participants discussed a lack of teamwork surrounding injury care and suggested that multi-professional educational courses could improve this by bringing together prehospital providers with nurses and physicians in-hospital.

Participants discussed greater involvement of the public in trauma education and prevention. Priorities included greater education on trauma prevention including road safety at crosswalks, on bicycles, and on motorcycles. They suggested teaching the basics of first aid to the public and especially motorcycle taxi drivers, who can quickly mobilize and support injured victims. Additionally, it was recommended that first aid kits should be distributed to motorcycle drivers and installed on public buses for no cost. Challenges cited included concerns over who would pay for the kits and how they could be restocked. Great concern was expressed about the improper use of motorcycle helmets by many riders, particularly female riders worried about their hair. Possible interventions included broader education about the proper fit and placement of helmets. Finally, participants discussed that despite a robust and strictly enforced motorcycle helmet law in Rwanda, a majority of riders seem to use them improperly.

Several research priorities were discussed by participants including increased national emphasis on publishing trauma and injury statistics and most suspected that the best method would be to establish a national trauma registry.

Both prehospital and in hospital providers discussed difficulties with the prehospital service. Suggestions to improve the service included the use of GPS coordinates to locate patients more quickly, as well as a mechanism to more easily discuss recommendations with those at the scene waiting for assistance. They noted issues with a limited central dispatch and opportunities for improvement in response times by allowing ambulances to bypass traffic to reach their destinations more quickly.

In-hospital improvement priorities highlighted issues with referral procedures from district hospitals and delays to access appropriate and definitive care in emergency departments. Additionally, issues with the lack of emergency departments in private hospitals were discussed. The importance of updating infrastructure to accommodate newer technologies was affirmed.

Similar conferences have been held to represent East Africa, Sub-Saharan Africa, Cape Town and Tanzania [15,23,24]. The Sub-Saharan Africa Emergency Care Consensus Conference recommendations paralleled many that we made in Rwanda, such as educating and empowering communities surrounding emergency health care, timely access to acute care diagnostics, the need for emergency clinical practice guidelines, and regionally appropriate courses for care [23]. In Tanzania no formal guidelines were released, but their symposium included Basic and Advanced Life Support and Pediatric Emergency Care Training workshops emphasizing the importance of educating trauma providers [24].

## CONCLUSIONS

The First National Rwanda Trauma Symposium drew together a diverse set of participants from across Rwanda who are working in the field of trauma. Attendees had productive discussions on how to address the rising burden of trauma in Rwanda. They presented current initiatives in the area of study and debated their recommended priorities for additional resources, education, and support towards trauma prevention. Finally, participants developed recommendations for policymakers and partners on the best way to move this agenda forward. In Rwanda, this symposium served to organize and familiarize a diverse group of partners with each other. Beyond Rwanda, we authors hope that this paper serves as an inspiration and a guide for other LMICs to pursue the best way to address their own increasing burdens of trauma.

**Acknowledgements/Ethics Approval:** All participants at the Rwanda National Trauma Symposium agreed to have their opinions and information included in the manuscript.
